# Long Low-Loss-Litium Niobate on Insulator Waveguides with Sub-Nanometer Surface Roughness

**DOI:** 10.3390/nano8110910

**Published:** 2018-11-06

**Authors:** Rongbo Wu, Min Wang, Jian Xu, Jia Qi, Wei Chu, Zhiwei Fang, Jianhao Zhang, Junxia Zhou, Lingling Qiao, Zhifang Chai, Jintian Lin, Ya Cheng

**Affiliations:** 1 State Key Laboratory of High Field Laser Physics, Shanghai Institute of Optics and Fine Mechanics, Chinese Academy of Sciences, Shanghai 201800, China; rbwu@siom.ac.cn (R.W.); qijia@siom.ac.cn (J.Q.); chuwei@siom.ac.cn (W.C.); jhzhang@siom.ac.cn (J.Z.); qiaolingling@siom.ac.cn (L.Q.); jintianlin@siom.ac.cn (J.L.); 2 University of Chinese Academy of Sciences, Beijing 100049, China; 3 State Key Laboratory of Precision Spectroscopy, East China Normal University, Shanghai 200062, China; mwang@phy.ecnu.edu.cn (M.W.); jxu@phy.ecnu.edu.cn (J.X.); zwfang@phy.ecnu.edu.cn (Z.F.); 52180920026@stu.ecnu.edu.cn (J.Z.); zfchai@phy.ecnu.edu.cn (Z.C.); 4 XXL—The Extreme Optoelectromechanics Laboratory, School of Physics and Materials Science, East China Normal University, Shanghai 200241, China; 5 Collaborative Innovation Center of Extreme Optics, Shanxi University, Taiyuan 030006, Shanxi, China

**Keywords:** lithium niobate, waveguide, photonic integrated circuit, propagation loss, optical lithography, chemo-mechanical polishing

## Abstract

In this paper, we develop a technique for realizing multi-centimeter-long lithium niobate on insulator (LNOI) waveguides with a propagation loss as low as 0.027 dB/cm. Our technique relies on patterning a chromium thin film coated on the top surface of LNOI into a hard mask with a femtosecond laser followed by chemo-mechanical polishing for structuring the LNOI into the waveguides. The surface roughness on the waveguides was determined with an atomic force microscope to be 0.452 nm. The approach is compatible with other surface patterning technologies, such as optical and electron beam lithographies or laser direct writing, enabling high-throughput manufacturing of large-scale LNOI-based photonic integrated circuits.

## 1. Introduction

Photonic integrated circuits (PICs) have shown the potential for use in complex information processing systems employing both quantum and classical light sources [1,2]. To increase computational efficiency and reconfigurability, PIC-based optical computers/calculators must have low propagation loss, fast tunability, and efficient optical interfacing. Currently, several materials have been utilized to construct large-scale PICs, including silicon and some semiconductor materials [3,4,5,6], fused silica [7,8], and bulk lithium niobate (LN) [9,10]. The advantage of silicon-based PICs is the high refractive index of silicon that enables the fabrication of compact light circuits with strong confinement and tight bends. In addition, the lithographic technology for high precision patterning of silicon and semiconductors is mature. However, silicon-based PICs intrinsically suffer from a relatively high propagation loss and a transmission window prohibitive for visible and shorter wavelengths. PICs can be built on fused silica and bulk LN crystals by local modification of the refractive index via either illumination of light or ion doping. Unfortunately, the refractive index increases achieved using these approaches are usually in the order of 10^−3^ to 10^−2^, resulting in large PICs footprints being required for minimizing the bending loss. Most importantly, the typical propagation losses of waveguides in state-of-the-art PICs are typically in the order of 10^−1^ dB/cm or higher, which ultimately limits the performance of PIC-based optical computers.

A revolutionary approach for building high-performance PICs has been emerging, enabled by the successful application of high quality lithium niobate on insulator (LNOI) nanophotonic structures. The first experimental proof of this approach was provided by first patterning the LNOI into the designated geometries using a femtosecond laser. The draft structures obtained after the femtosecond laser patterning, which has a relatively high sidewall roughness in the order of tens of nanometers, were then polished with a focused ion beam (FIB) milling to smoothen the sidewall [11]. This concept was soon incorporated into other lithographic technologies, such as optical lithography and electron beam writing (EBW), for defining the planar patterns on LNOI substrates followed by reactive ion etching to complete the nanostructuring of the LNOI [12,13]. The initial focus was mainly on optical microresonators [11,12,13,14,15,16,17,18,19,20,21], and other devices (such as waveguides and photonic crystals [22,23,24,25,26,27,28]) appeared shortly, taking advantage of the high surface smoothness of the sidewalls as a result of the ion dry etching. So far, the propagation loss in the LNOI waveguides has reached 0.04 dB/cm, highlighting their potential for use in large-scale PIC applications [29].

Notably, the ion etching step, which is necessary for achieving high quality sidewalls on LNOI nanophotonic structures, leaves a low but non-negligible surface roughness that is difficult to completely remove [29]. Moreover, the use of FIB or EBW in the patterning of LNOI makes the approach impractical for fabricating large-scale PICs due to their low throughputs and limited range of motion. Recently, we developed a technique for fabricating high-quality optical microresonators on LNOI with a quality factor above 10^7^ [30]. Since this technique does not involve an ion beam etching process, surface smoothness beyond that allowed by ion beam etching can be readily achieved, and the footprint sizes of PICs can be increased by patterning the LNOI photonic structures with either laser direct writing or optical lithography. Here, we experimentally show that we are able to realize 10-cm-long LNOI waveguides with a propagation loss of 0.027 dB/cm, which benefited from the low surface roughness of 0.452 nm measured with an atomic force microscope (AFM). The low loss waveguides can be essential building blocks for light modulation, beam delivery and manipulation, nonlinear optics, and optical signal processing.

## 2. Materials and Methods

**Figure 1:**
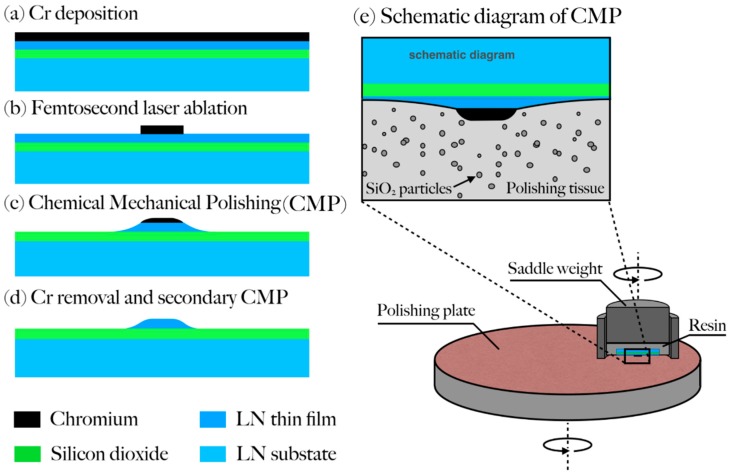
(**a**–**d**) Flow-chart of fabrication of lithium niobate on insulator (LNOI) waveguide and (**e**) schematic diagram of chemo-mechanical polishing (CMP).

A commercially available X-cut LNOI wafer fabricated by ion slicing (NANOLN, Jinan Jingzheng Electronics Co., Ltd., Jinan, Shandong, China) was chosen in our experiment as the material upon which the LNOI waveguides were produced [31]. The LN thin film had a thickness of 400 nm and was bonded to a SiO_2_ layer 2-μm-thick, which was grown on a LN substrate. The fabrication procedures are schematically illustrated in Figure 1, including (1) deposition of a thin layer of chromium (Cr) with a thickness of 600 nm on LNOI by magnetron sputtering and (2) patterning of the Cr film using femtosecond laser ablation. It is critical to carefully choose the pulse energy of the femtosecond laser to ensure the complete removal of the Cr film without damaging the LNOI underneath, which is enabled by the unique characteristics of the interaction of femtosecond laser pulses with various types of materials [32]. More details on the laser parameters can be found in Wu, R., et al. [30]. To produce a tightly focused spot ~1 μm in diameter, a 100× objective lens (M Plan Apo NIR, Mitutoyo Corporation, Kawasaki, Kanagawa, Japan) with a numerical aperture (NA) of 0.7 was used in our experiment. Femtosecond laser direct writing was conducted by translating the LNOI sample with a computer-controlled XY motion stage (ABL15020WB and ABL15020, Aerotech Inc., Pittsburgh, PA, USA, translation resolution ~100 nm). The focus of the laser beam was controlled in the Z direction using another one-dimensional stage with a translation resolution of 100 nm (ANT130-110-L-ZS, Aerotech Inc., Pittsburgh, PA, USA) on which the objective lens was installed. A charged coupled device (CCD) was installed above the objective lens for monitoring the fabrication process. The laser power was chosen to ensure the complete removal of the Cr thin film while keeping the LNOI underneath the Cr film intact. The patterned Cr disk served as a hard mask in the subsequent CM polishing.

The CM polishing was carried out using a wafer polishing machine (NUIPOL802, Hefei Kejing Materials Technology Co., Ltd., Hefei, Anhui, China). More details on CM polishing can also be found in Wu, R, et al. [30]. Note that the Cr film has a higher hardness than the LNOI, so LNOI would be completely removed by CM polishing, unless it is protected by a Cr mask. Finally, the Cr mask was removed by immersing the fabricated sample in a Cr etching solution (Chromium etchant, Alfa Aesar GmbH, Haverhill, MA, USA) for 10 min. All the procedures up to this stage were the same as those used for fabricating a high-Q microresonator, as described by Wu, R., et al. [30], For fabricating low-loss waveguides, the sample underwent an additional CM polishing at a relatively low pressure with a shorter polishing duration to improve the smoothness of the upper surface of LN waveguide, as illustrated in Figure 1d.

**Figure 2:**
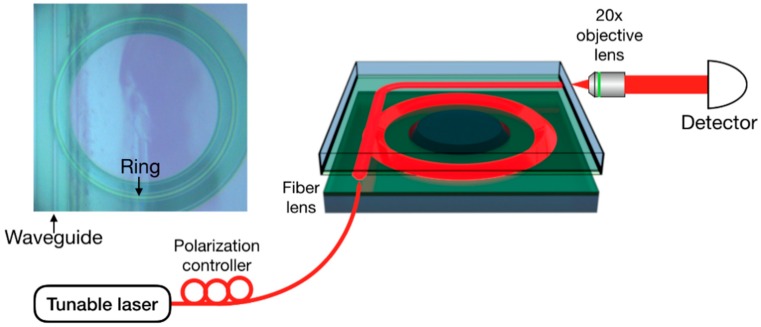
Schematic of the experimental setup for measuring the *Q* factor of the microring resonator. Left inset: Optical micrograph image of the microring resonator coupling with the waveguide, as indicated by the black arrows.

To characterize the propagation loss in the LNOI waveguide, we constructed a whispering gallery ring resonator through which the propagation loss of the LNOI waveguide was determined using *α* = 2π*n_eff_*/(*Qλ*), where α is the attenuation coefficient, *n_eff_* the effective refractive index, *Q* the quality factor of the ring resonator, and λ is the wavelength of the light beam. Both *n_eff_* and the *Q*-factor were determined from the transmission spectrum of microring resonator. The experimental setup for measuring the *Q* factor of the ring resonator is schematically shown in Figure 2. The light produced by a tunable laser (LTB-6728, Newport Corporation, Santa Clara, CA, USA) was coupled to a curved LNOI waveguide whose geometric parameters, including the thickness and width, were the same as that of the LNOI ring, determined with the use of a fiber lens. The exiting light was collected using a 20× objective lens (MPlanFL N, Olympus Corporation, Tokyo, Japan) into a detector (Model 1811, Newport Corporation, Santa Clara, CA, USA). The bend of the upper waveguide was intentionally introduced for preventing the stray light from the fiber laser from entering the objective lens located in front of the detector. The polarization direction of the light was adjusted with a fiber polarization controller. The curved waveguide was coupled to the LNOI ring resonator via evanescent coupling. Specifically, by carefully adjusting the distance between the coupling waveguide and the microring resonator, we were able to achieve the coupling condition crucial for obtaining an accurate intrinsic *Q* factor of the ring resonator. We fabricated five microrings and the measured optical losses were close to each other (with *Q* factors between 10^6^ and 10^7^), indicating that the fabrication technique is reliable and reproducible.

## 3. Results

**Figure 3:**
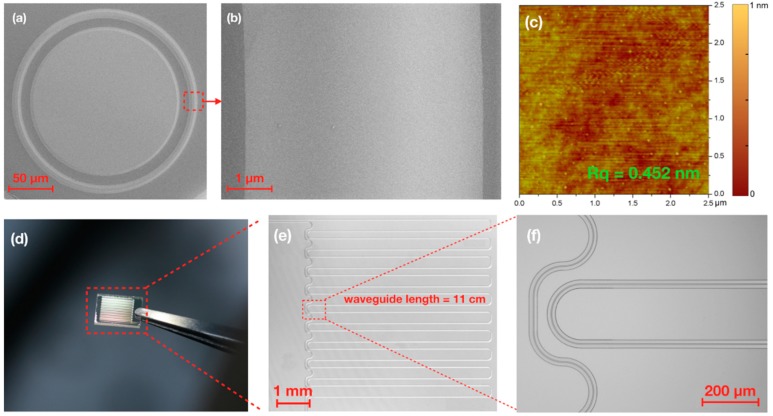
(**a**) Top-view scanning electron microscope (SEM) image of a lithium niobate (LN) microring resonator; (**b**) Zoomed view of the ridge of the microring resonator in (**a**); (**c**) Atomic force microscope (AFM) image of the ridge; (**d**) Picture of a chip consisting of an 11-cm-long waveguide captured by digital camera; (**e**,**f**) Zoomed images of the waveguides on the chip captured with an optical microscope.

Figure 3a shows the top-view scanning electron microscope (SEM) images of a LN micro ring resonator with a diameter of 160 μm. The width of the waveguide was ~3 μm. The close-up-view SEM of an arc of the ring highlights the high surface smoothness of the CM polished sample. A further atomic force microscope (AFM) inspection, as illustrated in Figure 3c, confirmed that a surface roughness *Rq* as low as 0.452 nm was achieved. The same fabrication technique was also used to fabricate a continuous 11-cm-long optical waveguide, as shown by the digital-camera-captured picture in Figure 3d, with details provided in the zoomed optical micrographs in Figure 3e–f. The total time of femtosecond laser ablation for fabricating the 11-cm-long waveguide was 90 min. At this moment, the footprint size of the PICs was only limited by the LNOI wafer size. The LNOI wafer can be made larger without much difficulty [33].

**Figure 4:**
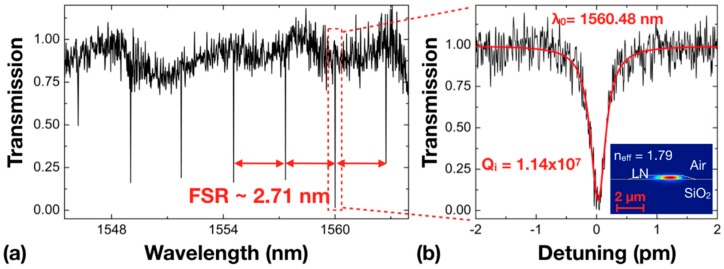
(**a**) Transmission spectrum of the LN microring resonator; (**b**) The Lorentz fitting (red curve) reveals a loaded *Q* factor of 5.70 × 10^6^, corresponding to an intrinsic *Q* factor of 1.14 × 10^7^. Inset: The optical mode distribution and *n_eff_* in the ring waveguide calculated using finite-difference time-domain (FDTD) simulation.

The optical loss characterization was performed using whispering-gallery-resonator-loss measurements. The propagation loss α is related to the *Q* factor of the ring resonator. Figure 4a shows the measured transmission spectrum for the wavelength range between 1546 and 1564 nm. The free spectral range (FSR) of the microring resonator was determined to be 2.71 nm, which is consistent with the 160 μm diameter of our ring resonator. The resonant lines appeared regularly spaced, indicating that mostly low-order modes exist in the ring resonator. One of the whispering-gallery modes at the resonant wavelength of 1560.48 nm was chosen for the measurement of the loaded *Q* factor by fitting with a Lorentz function, which reached 5.70 × 10^6^, corresponding to an intrinsic *Q* factor of 1.14 × 10^7^ in the critical coupling regime as evidenced in Figure 4b. The effective refractive index, *n_eff_* = *λ*^2^/(2π*R*·*FSR*), with a ring radius *R* of 80 μm and wavelength of 1560.48 nm, was calculated to be 1.79, which is in good agreement with our finite-difference time-domain (FDTD) simulation result given in the inset of Figure 4b. Combining the effective refractive index and the *Q*- factor obtained from the transmission spectrum, the propagation loss in the microring resonator was calculated to be 0.027 dB/cm using the aforementioned expression α = 2π*n_eff_*/(*Qλ*). This result represents the upper limit of the propagation loss in the LNOI waveguide fabricated using our method.

## 4. Discussion

The low propagation loss of 0.027 dB/cm was a result of the low surface roughness *Rq* of 0.452 nm on the fabricated LNOI waveguides. The surface roughness was improved using our technique in which the LNOI is purely patterned by the chemo-mechanical polishing without any use of ion beam etching [30]. The ion beam etching inherently leaves a small amount of surface roughness on the nearly vertical sidewalls, which is difficult to completely remove by top surface polishing [34,35]. This is the major reason that we were able to obtain a propagation loss lower than the waveguides fabricated by FIB or reactive ion etching. In the current experiment, we used a femtosecond laser to pattern the Cr hard mask. Generally speaking, the sidewall roughness on the Cr mask patterned by femtosecond laser ablation should be higher than the surface roughness of the LNOI photonic structures produced by ion beam etching. However, the sidewall roughness on the Cr mask only transfers to the underneath LNOI near the top surface, thus it can be completely suppressed with an additional polishing process for thinning the LNOI substrate after the removal of the Cr mask (Figure 1d).

Notably, the propagation loss obtained by measuring the *Q* factor of the ring resonator may have been underestimated for the straight segments in the LNOI waveguides, as presented in Figure 3d–f, due to a higher radiative loss in the ring resonator. Ultimately, the propagation loss of the LNOI waveguides is limited by the absorption in crystalline LN, which is well known to be in the order of ~10^−3^ dB/cm. Our measured loss was still one order of magnitude away from the theoretical limit, which could be attributed to several factors, including the radiative loss in the ring resonator and some unknown contamination on the surface of the LNOI waveguide, as the measurements were all carried out in a low-class clean room, existence of absorptive defects in the LNOI substrate owing to the imperfect crystal growth, and the remaining surface roughness left by the chemo-mechanical polishing process. Thus, to realize LNOI waveguides with propagation losses in the order of 1 × 10^−3^ dB/cm, many refinements should be systematically investigated in the future.

The fabrication resolution of femtosecond laser direct writing is typically in the order of 1 μm for inorganic materials such as glass, semiconductors, and metals. However, today’s optical lithography can easily achieve sub-micron or even 100-nm-level patterning resolutions. This should be sufficient for fabricating single-mode LNOI waveguides of narrower widths by which PICs, such as Mach-Zender interferometers and polarization convertors, can be built. The mode field of the LNOI waveguides can be tuned by coating them with fused silica for suppressing higher order modes as well as scattering loss due to the reduced contrast of the refractive index between the LNOI waveguide and the cladding environment [36]. With these improvements, the LNOI waveguides will become a major building block for PIC applications.

## 5. Conclusions

In this study, we achieved a propagation loss of 0.027 dB/cm in LNOI waveguides fabricated by combining femtosecond laser micromachining for patterning the Cr mask and chemo-mechanical polishing for transferring the laser-written patterns to the LNOI beneath the Cr mask. By eliminating the low-throughput FIB or EBW process, our technique enables the rapid fabrication of longer low-loss optical waveguides, which are only limited by the range of motion of the air bearing stage and the size of the LNOI wafers. Thus, our approach promotes the fabrication efficiency and reduces the cost of manufacturing LNOI PICs. The waveguides and ring resonators can be adopted for constructing complex PICs, enabling the mass production of LNOI PICs for optical communication and computation applications.
